# Motives for withdrawal of participation in biobanking and participants’ willingness to allow linkages of their data

**DOI:** 10.1038/s41431-021-00997-5

**Published:** 2021-11-22

**Authors:** Reinder Broekstra, Judith L. Aris-Meijer, Els L. M. Maeckelberghe, Ronald P. Stolk, Sabine Otten

**Affiliations:** 1 Department of Genetics, University Medical Center Groningen, University of Groningen, Groningen, the Netherlands; 2 Department of Epidemiology, University Medical Center Groningen, University of Groningen, Groningen, the Netherlands; 3 Department of Social Psychology, Faculty of Behavioral and Social Sciences, University of Groningen, Groningen, the Netherlands; 4 Institute for Medical Education, University Medical Center Groningen, University of Groningen, Groningen, the Netherlands

**Keywords:** Social sciences, Genetics, Medical research

## Abstract

Data repositories, like research biobanks, seek to optimise the number of responding participants while simultaneously attempting to increase the amount of data donated per participant. Such efforts aim to increase the repository’s value for its uses in medical research to contribute to improve health care, especially when data linkage is permitted by participants. We investigated individuals’ motives for participating in such projects and potential reasons for their withdrawal from participation in a population-based biobank. In addition, we analysed how these motives were related to various characteristics of the participants and their willingness to permit data linkage to their personal data for research. These questions were explored using a sample of participants in the Dutch Lifelines biobank (*n* = 2615). Our results indicated that motives for participation and withdrawal were premised on benefits or harm to society and to the individuals themselves. Although general values and trust both played key roles in participation, potential withdrawal and willingness to permit data linkage, they were differentially associated with motives for participation and withdrawal. These findings support and nuance previous findings by highlighting the distinctiveness and complexity of decision making regarding participation in or withdrawal from data donation. We suggest some new directions for improving recruitment, retention and safeguarding strategies in biobanking. In addition, our data provide initial evidence regarding how factors may relate with the probability that individuals will agree to data linkages, when controlling for their unique effects. Future research should further investigate how perceptions of harm and benefits may influence decision making on withdrawal of participation.

## Introduction

Continuous and full participation of members of the public in data repositories in the medical field is essential for effective scientific research. In addition, it creates the opportunity to improve health care for individuals by data enhancement [1–3]. To understand complex relationships in retaining health or developing disease, a large number of participants contributing large amounts of data is needed to get sufficient statistical power [3, 4]. Analyses on a large-scale, centralised data repository enable researchers to understand specific characteristics and behaviour on an individual level in an unprecedented way, which can impact both research and clinical practice [4]. In addition, advances in information technology create the possibility to analyse vast amounts of genetic (genomic) data, and the potential to understand more about risk factors for developing diseases or the course of the disease. As a result of these new possibilities for analysing and knowledge building, the quest to advance the collection and use of data from both patients and healthy volunteers has become urgent, further increasing the efforts on exponential growth of data repository and international data-sharing or linkage projects [5, 6].

Large-scale, centralised data repositories can be large prospective population-based cohort studies and biomedical biobanks. These biomedical cohort studies and biobanks (further referred to as biobanks) store large quantities of biological specimens as well as data extracted from questionnaires and measurements as a resource for research, while being managed by professional standards [7, 8]. Biobanks and similar data repositories seek to optimise the number of responding participants increasing the data repositories’ value for health care and research, while simultaneously attempting to increase the amount of data donated per participant. Yet recruiting participants in biobanking in Europe is a challenge even in ‘willing’ populations, for example, among Finnish people [9]. Achieving high participation rates will most likely become an even larger challenge, considering the development of participation rates in population-based survey studies in the past 35 years [10, 11]. In light of these challenges for recruiting new participants, retainment of current participants should be a growing concern for those responsible for data repositories and for those using them.

The establishment of mutually enhancing data linkages between existing data repositories might be an important solution for improving the collection and use of data. Data linkage could provide multiple opportunities, such as providing unique insights in existing data without significant efforts of participants [5], or enabling individually tailored insights from data that might be communicated in return for participation to benefit a participants’ health directly [3]. As a result, data linkage might influence current rates of participation and data density, especially when individually tailored results are returned or accessible as return of an individual benefit.

Yet data linkage is challenging when it comes to protection of personal sensitive data, and promising potential individual benefits [1, 5]. The clinical utility of individually tailored insights from research is not straight forward and an ongoing topic of debate, for example, the clinical utility of individuals’ polygenic risk scores for various illnesses [12] or of data from wearables [13]. Research has shown that motives for non-participation and (potential) withdrawal are linked to issues regarding commercialisation, confidentiality and privacy of data [14]. Simultaneous, biobank’s operating procedures, such as restricted data access policies, or its decision to return findings from research are associated with higher participation levels [15, 16].

Achieving optimised rates of participation therefore requires a sound understanding of citizens’ decisions to participate or not-participate in biobanks. Especially understanding decisions of withdrawal might help to understand differences between participant and (potential) non-participant. Subsequently, it provides valuable insights in the viability of data sharing as potential solution for optimised rates. Yet information about rates of withdrawal in biobanking is scarce, or not publicly available, which limits current understanding of mechanisms of withdrawal. Although one Swedish study investigating withdrawal rates in biobanking during 2005 and 2006 indicated low withdrawal rates at that time [17], the potential of data and data linkage has rapidly developed, and concerns about data linkage in the last decade became realities [18].

So far, studies have pointed to the key roles and interplay of individuals’ characteristics in their participation and non-participation in biobanking. For example, a variety of characteristics on an individual level have been found to be positively associated with participation in biobanks, such as a higher educational attainment [19–21], positive attitudes towards research and society [6, 20, 22], a general concern about others [23] and high levels of societal or social trust [6, 21]. In addition, several studies suggest that prosocial attitudes and trust in others are especially important factors that independently relate to positive decisions regarding participation [24, 25].

Some of these characteristics may also be positively related to withdrawal of participation, depending on the specific context of the biobank, e.g., studies in Europe, United States and Australia showed that concerns about privacy and confidentiality were robustly related to lower willingness to participation [6, 26, 27]. Specifically these concerns could be triggered in case of data linkage or access, especially when commercial enterprises are involved [28, 29]. An Australian study showed that higher educational attainment was associated with a stronger reduction of trust in a public biobank that allows access to third parties [30]. Other studies found that those concerns about privacy or confidentiality were associated with anxiety for commodification of contributed samples or data [31, 32]. Furthermore, technologies are perceived differently depending on health expectancies, gender or social trust [33]. For example, men perceive fewer risk and more benefits in gene technology than women [34]. Levels of trust in data linkage or commercial enterprises can also inhibit or decrease perceived risk in participation, which might be decisive for continuation or withdrawal [21, 30].

Hence, the contextual and procedural characteristics of biobanks can affect participation via withdrawal, depending on the characteristics of (potential) participants. Changes in the data after withdrawal can lead to bias in the data and the analysis of the data from data repositories, especially in biobanks representing populations of both participants and non-participants, which could be reduced by refinement of the data [25, 35]. While previous studies have broadly explored the characteristics and motives of participants and non-participants in biobanks as well as in data sharing [6, 20, 21, 26, 36, 37], further research on the associations between individual participants’ characteristics and their motives for participation and withdrawal would be beneficial [35]. In particular, research on the associations between participants’ motives of withdrawal and their psychological characteristics could yield insights into how contextual biobanking elements could impact on individuals’ continued participation or their withdrawal [24, 25].

An investigation of the drivers of participation and withdrawal behaviour is therefore required to reduce potential biases. This study aimed to gain insight in the possibilities to classify individuals who participate in population-based biobanks according to various demographic and psychological key characteristics, and find distinguishing traits between those participants who are more likely to withdraw their participation and those who are not. In addition, we tried to identify factors that will affect the likelihood that participants would accept the linkage of their personal data for research purposes. The study could then yield insights for optimising recruitment and retainment strategies associated with biobanking or similar data repository initiatives, while preventing withdrawal of participation.

## Materials and methods

### Procedure and participants

We administered an online survey in August 2018 within a randomly selected sample of 2615 respondents, who were among the 167,000 participants of Lifelines biobank. Lifelines is a population-based biobank and large, multigenerational prospective cohort study in the Northern part of the Netherlands [7]. All Lifelines participants consented between 2006 and 2013 to participate in the biobank, allowing it to examine 10% of the population of Northern part of the Netherlands. It does not offer incentives for research participation. Lifelines applies a broad range of investigative procedures for assessing biomedical, sociodemographic, behavioural, physical and psychological factors, with a particular focus on multi-morbidity and complex genetics.

At the time of data collection for the current study, our respondents were registered as active participant of Lifelines, which implies they had not withdrawn their participation at the time of filling in our survey and were actively participating in the biobank. The response rate for the invitation of this additional study was 22.2%. We stratified our sample to improve its representativeness compared to the representativeness of the Lifelines cohort for the Dutch population [7, 38]. We found that our study sample, stratified by sex (male = 50.5%) and age (*M* = 56, SD = 15.88), nevertheless has more individuals with a high educational level, registered partner and good self-reported health compared to the Lifelines population [38].

### Measures

#### Demographic characteristics and self-reported health

We measured demographic characteristics that were previously associated with participation or willingness to participate in a biobank, such as marital status [21], education level [19, 24], religion [16, 36], residential area [38] and self-reported general health *(1 = ‘very poor’, 5 = ‘very good’)* [19, 39]. Work status was included as an additional indicator of socio-economic status.

#### Prosocial intrapersonal characteristics

##### Prosocial orientation

We applied two measures to determine respondents’ general orientations towards other individuals within their behaviour, which we refer to as prosocial orientation. The first was their actual prosocial behaviour reflected in their organ and blood donor status and the frequency of charitable donations. The second was the degree to which respondents cared about other individuals’ outcomes in choices concerning resource allocation, which were measured using six items in the social value orientation (SVO) scale. This scale is designed to measure the magnitude of individuals’ general concern for others [40]. Replicating previous research, our results indicated excellent reliability with Cronbach’s alpha >0.90 [41].

##### Values

We used 11 items based on the Theory of Basic Values from Schwartz to assess motivations of and beyond self-interest as guiding principles in individuals’ life [42]. For example, about the relevance of individual pleasure in life as guiding principle or the relevance of health and health care. Although our primary focus was on motivations of and beyond self-interest in the health care context, we investigated these motivations relating to a wider context, such as natural environmental values [43]. As such, values can provide more information about both magnitude and direction of individuals’ motives of self-interest and beyond. We applied an adapted version of the Environmental Personal Values Questionnaire (E-PVQ) scale [44]. We used two different subscales from the E-PVQ to measure hedonic values associated with (individual pleasure) (e.g., ‘it is important [for him/her] to have fun’) and biospheric values relating to the natural environment (e.g., ‘it is important [for him/her] to protect the natural environment’). In addition, we used a tailored subscale on healthspheric values relating to health and health care concerns (e.g., ‘it is important [for him/her] to live a fit life’). This subscale was designed in collaboration with the developers of the E-PVQ. The scales’ reliability was good, with Cronbach’s alpha >0.80 for all of the subscales, thus confirming reliability [44].

##### Societal trust

We identified trust as a key factor of participation and non-participation in biobanking. This trust can relate to the general trust in society and a domain-specific trust in research, especially in case of data linkage in research. In addition, a previous study distinguished levels of trust in organisations from levels of trust in employees of these organisations [45]. We therefore investigated both the level of individuals’ trust in society, and the level of trust in research organisations, in particular their data management and handling (hereinafter referred to as ‘DM&H’). The latter should automatically address issues about privacy in data management and handling. First, societal trust was measured using the trust-based framework proposed by Mayer et al. [46]. We used six items reflecting trust in the government and in other citizens that were measured with a 5-point Likert scale (1 = ‘strongly disagree’, 5 = ‘strongly agree’). Examples included ‘the government acts with good intentions’; ‘people in the Netherlands are willing to help each other’ and ‘people are trustworthy’. These items focused on aspects of societal or sometimes called social trust, namely trustworthiness, good intentions and competence. The combined scale with all six items demonstrated good reliability (Cronbach’s alpha = 0.86).

Second, we used five similar 5-point Likert scale items focusing on trust in the DM&H of different types of research organisations (hospitals, governmental institutes, universities, large-sized commercial enterprises and small and medium-sized enterprises). An illustrative item was ‘I believe that the hospital correctly, adequately, and fairly stores and treats my personal data’. Besides measuring trust in organisations as entities, we also measured trust in specific research employees using four items (hospital researchers, university researchers, commercial market researchers and commercial polling researcher). An illustrative item was ‘I trust employees of commercial polling enterprises to conduct research using a correct, fair and careful approach’.

We conducted a principal component analysis with an oblimin rotation given several high intercorrelations between several research items (*r* > 0.50). The results of the analysis revealed that the items could be reduced to three factors explaining 71.90% of the total variance. The first factor, comprising four items, was trust in DM&H of the government (organisations and their employees) and explained 45.74% of the total variance (*α* = 0.83). The second factor, comprising two items, was trust in the DM&H of commercial enterprises and explained 14.24% of the total variance (*r* = 0.65*)*. The third factor, trust in DM&H of commercial researchers, comprised two items and explained 11.92% of the total variance (*r* = 0.47*)*. Correlations between the components were low to moderate (*r* < 0.40).

#### Motives for participation and potential withdrawal

Although we included values to assess motivations of and beyond self-interest, a better understanding of motives would require including the biobanking context-specific motives. We measured therefore a variety of potentially relevant motives for participation and withdrawal in biobanking derived from an earlier explorative qualitative study on this topic [45]. These 5-point Likert scale items about motives included topics of potential benefits and potential harms of participation in biobanking. We performed a factor analysis of nine motives for participation (e.g., ‘I contribute to health care by participating’) and seven motives for potential withdrawal (e.g., ‘I had a negative experience during a visit’). We conducted two distinct principal axis factor analyses with oblimin rotation, which revealed that motives for participation and withdrawal could be clearly differentiated according to two factors. Yet, three of our initial items about potential motives were excluded for further analyses due to their low communalities (<0.30). The appropriateness of these factors relating to remaining motives for participation and withdrawal was confirmed by the results of Kaiser–Meyer–Olkin tests (0.795 and 0.785) and Bartlett’s tests of sphericity (*χ*^2^(120) = 13,557.87, *p* < 0.01; *χ*^2^(21) = 5025.84, *p* < 0.01), respectively. The two factors relating to participation, that is, ‘societal benefits’ and ‘individual benefits’, respectively, explained 45.19% and 17.91% of the total variance. The two factors relating to potential withdrawal, that is, ‘societal harm’ and ‘individual harm’, respectively explained 37.06% and 8.89% of the total variance.

### Willingness of participants to allow personal data linkages

We measured the participants’ willingness to accept data linkage of five types of potentially relevant personal data (e.g., medical, financial, and sensor data) for large-scale scientific research using a 5-point Likert scale. Data linkages were first introduced to the participants with several illustrations of their application, for example, in electronic health records or annual tax income report. Next, participants were asked the following question: ‘How likely would it be for you to share the following data for large-scale scientific research?’ (1 = ‘very unlikely’, 5 = ‘very likely’). The total score ranged from 5 to 25 and served as an indicator of participants’ willingness to agree to the linking and sharing of their personal data to a biobank.

### Data analysis

We calculated descriptive statistics for demographic and psychological variables (motives and prosocial orientations). We applied *χ*^2^ tests, (M)ANOVA tests or independent *t*-tests to calculate potential differences in means for motives to participate or withdraw among different demographic groups. Furthermore, we performed bivariate correlational and linear multiple regression analyses, applying a manual entry strategy to investigate associations between motives and individual characteristics. We entered the variables forward block wise following the order of our description of measurements. The statistical power of this sample was high (1.00), and an a priori conservative power analysis with Gpower software showed a minimum of *n* = 1347. All other analyses were conducted using the SPSS software, version 25.0 [47].

## Results

Before proceeding to our analyses, the quality of the data was assessed by explorative descriptive analyses of missing data. The assessment showed less than 5% missing data per variable. Even though there was some missing data, there was no clear pattern in its missingness. Hence, we believed that these data were missing at random. We proceeded with descriptive and explorative analyses with univariate comparisons. Subsequently, we conducted our main analyses of predicting motives of withdrawal and intention to allow participants’ data to be linked.

### Demographic and prosocial characteristics

Table 1 depicts the participants’ demographic and prosocial characteristics. Of the 2615 respondents, 79.5% had registered partnerships, such as marriages, and 55.8% had paid jobs. The participants reported being generally in good health, with a mean score of 3.97 on a 5-point scale. A total of 41% of the respondents were highly educated (*n* = 1083), and 37% were religious (*n* = 972). The majority of the participants (77%) lived in rural areas (*n* = 2019). Furthermore, 12% of the respondents were blood donors and 56% registered as organ donors. The mean value for contributions to charities was moderate at 2.68 on a 5-point scale. The SVO score for participants’ concern about others was 32.25, indicating a strong prosocial orientation [41]. In addition, Table 1 shows most participants scored higher for values relating to health, a healthy lifestyle and their own pleasure than for environmental values. They reported high levels of trust in the government and in other citizens (*M* = 3.25, SD = 0.63), and especially in the government’s DM&H (*M* = 3.55, SD = 0.81). However, they had lower levels of trust in the DM&H practices of commercial researchers (*M* = 3.32, SD = 0.80) and commercial enterprises (*M* = 3.14, SD = 0.82).

**Table 1:** Demographic characteristics and the self-reported health of respondents being participants in a Dutch population-based biobank.

	*N */ *M*	% / SD
*Age*		
<30 years	231	8.8%
31–40 years	252	9.7%
41–50 years	357	13.7%
51–60 years	580	22.2%
>60 years	1192	45.6%
*Marital status*		
Registered partner	2078	79.5%
No registered partner	536	20.5%
*Educational level*		
High	1083	41.4%
Moderate	938	35.9%
Low	559	21.7%
*Paid job*	1460	55.8%
*Religious*	972	37.2%
*Area of residence*		
Rural	2019	77.2%
Urban	537	20.5%
*Self-reported health*	3.97	0.71
*Frequency of donations to charities*	2.68	1.08
*Blood donor*	306	11.7%
*Organ donor*	1460	55.8%
*Social value orientation score*	32.25	14.19
*Values*		
Healthspheric	3.99	0.66
Hedonic	4.25	0.65
Biospheric	3.76	0.75
*Societal trust*	3.25	0.63
*TDM&H/government*	3.55	0.81
*TDM&H/commercial enterprises*	3.14	0.82
*TDM&H/commercial researchers*	3.32	0.80

The values in the table are either means (*M*) with standard deviations (SD) or frequency numbers (*N*) with percentages (%) relating to the total sample. All variables’ missing data <5%.

TDM&H denotes trust in data management and handling.

### Motives for participation and withdrawal

Table 2 provides the results of our analyses of motives for participation and potential withdrawal, respectively. Overall, respondents scored higher on societal than for individual motives relating to both participation (4.06 vs 2.92) and withdrawal (3.94 vs 3.49). Table 3 shows Pearson’s correlations between motives for participation and (potential) withdrawal and intrapersonal characteristics. We found modest associations of motives (*r* < 0.30), and especially participation motivated by societal benefits, with the various indicators of individual prosocial values and trust. All of the trust indicators were positively related to participation motivated by societal as well as individual benefits.

**Table 2:** Motives for participation in and potential withdrawal from a Dutch population-based biobank.

	*M*	SD	Loading
***Motives of participation—societal benefits***	4.06	0.50	(*α* = 0.83)
I contribute to the health of future generations by participating	4.23	0.59	0.74
I contribute to health care by participating	4.14	0.55	0.91
I contribute to science by participating	4.10	0.57	0.84
I contribute to society by participating	3.77	0.74	0.55
***Motives of participation—individual benefits***	2.92	0.80	(*α* = 0.84)
I learn about myself by participating	3.86	0.75	–
I perceive participation as moral duty	3.22	0.97	0.47
I have fun by participating	2.89	0.95	0.74
People I know participate	2.71	1.21	–
I feel proud by participating	2.65	0.99	0.96
***Motives of withdrawal—societal harm***	3.94	0.84	(*α* = 0.77)
The research organisation becomes a for-profit organisation	3.98	0.89	0.87
The research organisation starts active collaboration with commercial enterprises	3.91	0.93	0.83
The research does no longer contribute to health care	3.65	0.85	–
***Motives of withdrawal—individual harm***	3.49	0.64	(*α* = 0.61)
I have a negative experience during a visit	3.58	0.87	0.67
The research organisation gets negative news reports	3.51	0.83	–
De time investment becomes too high	3.48	0.91	0.59
The research aim changes	3.41	0.78	0.42

The values in the table are probability (*p*) with standard deviation (SD), and reliability statistic Cronbach’s alpha (*α*). Loadings of items with communalities <0.30 are not reported (–), and were left out in further analyses.

**Table 3:** Correlations.

Variable	1	2	3	4	5	6	7	8	9	10	11	12	13	14	15
1. Motives of participation—societal benefits	1														
2. Motives of participation—individual benefits	**0.38**	1													
3. Motives of potential withdrawal—societal harm	0.04^S^	**−0.12**	1												
4. Motives of potential withdrawal—individual harm	−0.05^S^	**−0.12**	**0.37**	1											
5. Age	**0.06**	**0.10**	**0.11**	0.04	1										
6. Self-reported health	0.03	−0.05^S^	**0.06**	0.03	**−0.07**	1									
7. Frequency of charitable donations	**0.11**	−0.03	**0.06**	0.02	**0.17**	0.05^S^	1								
8. Social value orientation (SVO) score	**0.08**	−0.03	**0.05**	−0.01	0.01	0.00	**0.13**	1							
9. Healthspheric values	**0.26**	**0.09**	**0.16**	**0.05**	**0.20**	**0.24**	**0.13**	**0.11**	1						
10. Hedonic values	**0.23**	0.05^S^	**0.11**	**0.05**	**−0.10**	**0.13**	0.04	**0.08**	**0.62**	1					
11. Biospheric values	**0.28**	**0.11**	**0.16**	0.02	**0.25**	**0.09**	**0.17**	**0.14**	**0.68**	**0.43**	1				
12. Societal trust	**0.15**	**0.05**	0.00	0.03	0.02	**0.22**	**0.16**	**0.12**	**0.18**	**0.11**	**0.15**	1			
13. TDM&H/government	**0.22**	**0.07**	0.04^S^	−0.02	−0.05^S^	**0.12**	**0.09**	**0.11**	**0.18**	**0.17**	**0.14**	**0.40**	1		
14. TDM&H/commercial enterprises	**0.06**	**0.20**	**−0.08**	**−0.06**	**0.06**	0.03	**0.07**	0.05^S^	**0.06**	0.02	0.02	**0.24**	**0.32**	1	
15. TDM&H/commercial researchers	**0.14**	**0.13**	−0.04^S^	−0.03	0.04^S^	**0.07**	**0.10**	**0.07**	**0.06**	0.02	**0.07**	**0.35**	**0.46**	**0.40**	1

**Bold** = significant with *p* < 0.01; ^S^ = significant with *p* < 0.05.

To obtain an overview of systematic associations of motives with demographic and psychological characteristics, we conducted an exploratory comparative analysis of means. Table 4 shows that motives relating to individual benefits as well as harm differed significantly yet modestly according to participants’ educational levels. Motives to participate for individual benefit were stronger among participants with lower education levels (*M* = 3.12, SD = 0.72) than among moderately educated (*M* = 2.98, SD = 0.81) and highly educated participants (*M* = 2.76, SD = 0.78). However, the reverse was true for motives to withdraw participation relating to individual harm. These motives were less important for participants with lower education levels (*M* = 3.45, SD = 0.65) and moderately educated (*M* = 3.49, SD = 0.62) than they were for highly educated participants (*M* = 3.54, SD = 0.54).

**Table 4:** A comparison of means obtained for respondents’ motives for participating in and potential withdrawing from a Dutch population-based biobank.

	Motives			
	Participation		Withdrawal	
	Societal benefits	Individual benefits	Societal harm	Individual harm
	*p* (Levene’s *F*), BF / *M* (SD)	*p* (Levene’s *F*), BF / *M* (SD)	*p* (Levene’s *F*), BF / *M* (SD)	*p* (Levene’s *F*), BF / *M* (SD)
***Age***	0.19 (1.50), n.a.	0.25 (1.33), n.a.	**<0.01** (**3.94**), **n.a.**	0.16 (1.60), n.a.
<30 years	4.03 (0.48)	2.90 (0.79)	3.52 (0.68)	3.49 (0.59)
31–40 years	3.97 (0.59)	2.84 (0.80)	3.62 (0.72)	3.49 (0.62)
41–50 years	4.03 (0.53)	2.84 (0.85)	3.77 (0.65)	3.55 (0.58)
51–60 years	4.08 (0.50)	2.84 (0.84)	3.85 (0.72)	3.49 (0.65)
>60 years	4.09 (0.47)	3.02 (0.75)	3.88 (0.75)	3.47 (0.66)
***Gender***	0.97 (0.00), 0.02	**<0.01** (**6.86**), **0.48**	**<0.01** (**19.16**), **220.37**	**<0.01** (**26.88**), **10 088.64**
Male	4.06 (0.51)	2.97 (0.80)	3.88 (0.90)	3.43 (0.67)
Female	4.06 (0.49)	2.88 (0.80)	4.03 (0.77)	3.56 (0.60)
***Marital status***	0.11 (2.50), 0.06	0.35 (0.88), 0.02	0.35 (0.86), 0.02	0.17 (1.91), 0.04
Registered partner	4.07 (0.50)	2.93 (0.80)	3.95 (0.83)	3.50 (0.63)
No registered partner	4.03 (0.51)	2.89 (0.81)	3.91 (0.88)	3.46 (0.66)
***Educational level***	0.20 (1.63), 0.00	**<0.01** (**44.54**), **3.97E+15**	**0.05** (**3.11**), **0.01**	**<0.01** (**11.59**), **39.92>**
High	4.08 (0.53)	2.75 (0.78)	3.99 (0.82)	3.56 (0.59)
Moderate	4.05 (0.47)	2.98 (0.77)	3.91 (0.84)	3.46 (0.66)
Low	4.04 (0.50)	3.12 (0.81)	3.90 (0.89)	3.41 (0.68)
***Paid job***	**<0.01** (**9.81**), **2.08**	**<0.01** (**19.83**), **303.72**	**<0.01** (**7.03**), **0.53**	0.33 (0.94), 0.03
Yes	4.03 (0.52)	2.86 (0.81)	3.91 (0.84)	3.50 (0.60)
No	4.10 (0.48)	3.00 (0.78)	3.99 (0.84)	3.48 (0.66)
***Religious***	0.09 (2.89), 0.07	0.24 (1.37), 0.03	0.15 (2.07), 0.04	**0.02** (**5.29**), **0.22**
Yes	4.04 (0.47)	2.94 (0.80)	3.91 (0.86)	3.45 (0.66)
No	4.07 (0.51)	2.91 (0.80)	3.96 (0.83)	3.51 (0.63)
***Area of residence***	0.87 (0.03), 0.02	0.53 (0.40), 0.02	0.13 (2.36), 0.05	0.22 (1.50), 0.03
Rural	4.06 (0.49)	2.93 (0.79)	3.96 (0.83)	3.50 (0.63)
Urban	4.06 (0.52)	2.90 (0.80)	3.90 (0.87)	3.46 (0.66)
***Blood donor***	0.28 (1.17), 0.03	0.52 (0.41), 0.01	0.84 (0.04), 0.02	**<0.01** (**6.36**), **0.38**
Yes	4.03 (0.56)	2.95 (0.82)	3.94 (0.79)	3.58 (0.66)
No	4.06 (0.49)	2.92 (0.80)	3.95 (0.85)	3.48 (0.64)
***Organ donor***	**<0.01** (**11.07**), **3.91**	**0.04** (**4.20**), **0.02**	0.26 (1.28), 0.03	0.06 (3.64), 0.04
Yes	4.09 (0.51)	2.89 (0.80)	3.96 (0.83)	3.47 (0.64)
No	4.03 (0.49)	2.96 (0.80)	3.92 (0.86)	3.52 (0.63)

The values in the table are either probability values (*p*) with Levene’s test F-statistic (Levene’s *F*) and Bayes Factor (BF) or means (*M*) with Standard deviations (SD).

**Bold** = significant with *p* < 0.01.

In addition, we found that gender had a significant effect on the relevance of certain motives; whereas women scored lower than men for participation motivated by individual benefits, they scored higher for motives to withdraw participation based on societal and individual harm. Furthermore, blood and organ donors differed in their participation and potential withdrawal motives. Blood donor status simply predicted a stronger motivation to withdraw participation to avoid individual harm, whereas organ donor status was associated with higher scores for societal benefits and weaker scores for individual benefits as motives for participation. Finally, job status was significantly associated with all motives except for those relating to individual harm. The other demographic characteristics (i.e., marital status, religiosity, and urban dwelling) were not significantly associated with motives for participation and withdrawal.

### Predicting motives for potential withdrawal

All of the respondents were participating in a biobank at the time of the study. However, this does not imply that their long-term participation was guaranteed. We therefore asked the respondents under what circumstances they would consider withdrawing from further participation. Accordingly, we performed linear multiple regressions to predict the importance of participants’ motives for withdrawal, considering demographic variables, motives for participation and prosocial psychological characteristics, while controlling for motives relating to either individual or societal harm (see Table 5). All assumptions for both multiple linear regression analyses were met: the normality p-plot and the scatterplot of standardised residuals showed that the data met the assumptions of normality, linearity and homogeneity of variance; there were no indications of multicollinearity (VIF scores <10.0; tolerance >0.1). Significant predictors of withdrawal based on anticipated societal harm were: a stronger motivation to benefit society via participation (*B* = 0.05), a weaker motivation to benefit individually via participation (*B* = −0.10), being older (*B* = 0.12), being female (*B* = 0.06), better self-reported health (*B* = 0.04), being an organ donor (*B* = 0.04), a higher SVO score (*B* = 0.04), stronger biospheric values (*B* = 0.08), lower levels of societal trust (*B* = –0.05), a high level of trust in the government’s DM&H (*B* = 0.08) and a low level of trust in commercial enterprises’ DM&H (*B* = −0.08). The total explained variance was 19%.

**Table 5:** Results of the linear regression of motives for withdrawal and data linkages.

		Predicting societal harm as motive of withdrawal			Predicting individual harm as motive of withdrawal			Predicting Intention to link personal data		
Variable	Step	Beta	95% CI unst. *B*	*p*	Beta	95% CI unst. *B*	*p*	Beta	95% CI unst. *B*	*p*
Withdrawal motive societal harm	1	**–**	**–**	**–**	**0.36**	[**0.24, 0.30**]	<**0.01**	**−0.08**	[**−0.10, −0.03**]	<**0.01**
Withdrawal motive individual harm	1	**0.35**	[**0.41, 0.51**]	<**0.01**	**–**	**–**	**–**	**−0.10**	[**−0.14, −0.06**]	<**0.01**
Participation motive societal benefits	2	**0.05**	[**0.01, 0.15**]	**=0.03**	−0.04	[−0.11, 0.00]	=0.06	**0.20**	[**0.21, 0.32**]	<**0.01**
Participation motive individual benefits	2	**−0.10**	[**−0.14, −0.06**]	<**0.01**	−0.04	[−0.07, 0.00]	=0.06	**0.05**	[**0.01, 0.08**]	**=0.01**
Age	3	**0.12**	[**0.00, 0.01**]	<**0.01**	−0.04	[−0.01, 0.00]	=0.18	**−0.13**	[**−0.01, 0.00**]	<**0.01**
Gender (Male = 1, female = 2)	3	**0.06**	[**0.03, 0.16**]	<**0.01**	**0.07**	[**0.04, 0.14**]	<**0.01**	**−0.07**	[**−0.14, −0.04**]	<**0.01**
Marital status (Without partner = 0, Partner = 1)	3	−0.03	[−0.14, 0.02]	=0.16	**0.05**	[**0.02, 0.14**]	**=0.01**	0.02	[−0.02, 0.10]	=0.27
Educational level (Low = 1, Moderate = 2, High = 3)	3	−0.03	[−0.03, 0.00]	=0.21	**0.07**	[**0.01, 0.04**]	<**0.01**	**0.09**	[**0.02, 0.05**]	<**0.01**
Paid job (no = 0, yes = 1)	3	0.02	[−0.05, 0.11]	=0.50	0.00	[−0.07, 0.05]	=0.79	−0.02	[−0.09, 0.04]	=0.42
Religious (no = 0, yes = 1)	3	0.01	[−0.04, 0.09]	=0.49	−0.03	[−0.09, 0.00]	=0.10	−0.02	[−0.08, 0.02]	=0.25
Residence (rural = 1, urban = 2)	3	−0.02	[−0.11, 0.04]	=0.36	−0.02	[−0.09, 0.02]	=0.21	0.02	[−0.03, 0.09]	=0.29
Self-reported health	3	**0.04**	[**0.00, 0.09**]	**=0.05**	−0.01	[−0.04, 0.03]	=0.62	0.04	[−0.01, 0.07]	=0.06
Frequency of charitable donations	4	−0.00	[−0.03, 0.03]	=0.96	0.02	[−0.01, 0.03]	=0.47	0.03	[−0.01, 0.04]	=0.14
Blood donor (no = 0, yes = 1)	4	−0.01	[−0.13, 0.06]	=0.48	**0.05**	[**0.02, 0.17**]	**=0.02**	0.03	[−0.01, 0.14]	=0.11
Organ donor (no = 0, yes = 1)	4	**0.04**	[**0.01, 0.14**]	**=0.03**	**−0.08**	[**−0.16, −0.06**]	<**0.01**	**0.07**	[**0.04, 0.14**]	<**0.01**
Social value orientation score	5	**0.04**	[**0.00, 0.00**]	**=0.05**	**−0.04**	[**0.00, 0.00**]	**=0.05**	−0.03	[0.00, 0.00]	=0.16
Healthsperic values	6	0.03	[−0.03, 0.08]	=0.41	0.02	[−0.03, 0.06]	=0.53	−0.05	[−0.08, 0.00]	=0.08
Hedonic values	6	0.02	[−0.03, 0.07]	=0.36	0.02	[−0.02, 0.05]	=0.39	0.00	[−0.04, 0.04]	=0.90
Biospheric values	6	**0.08**	[**0.02, 0.12**]	<**0.01**	−0.03	[−0.05, 0.02]	=0.29	0.01	[−0.03, 0.05]	=0.60
Societal trust	7	**−0.05**	[**−0.12, −0.01**]	**=0.02**	**0.07**	[**0.02, 0.11**]	<**0.01**	**0.09**	[**0.05, 0.14**]	<**0.01**
TDM&H/government	7	**0.08**	[**0.06, 0.20**]	<**0.01**	−0.04	[−0.10, 0.00]	=0.08	**0.08**	[**0.05, 0.16**]	<**0.01**
TDM&H/commercial enterprises	7	**−0.08**	[**−0.14, −0.04**]	<**0.01**	−0.02	[−0.06, 0.02]	=0.31	**0.14**	[**0.09, 0.16**]	<**0.01**
TDM&H/commercial researchers	7	−0.03	[−0.08, 0.02]	=0.22	0.00	[−0.04, 0.02]	=0.74	**0.06**	[**0.01, 0.09**]	**=0.01**
*F* (model 7)			25.30			21.02			27.54	
Adjusted *R*^2^			0.19			0.17			0.21	

*B* = regression coefficient, *CI* = confidence interval.

**Bold** = *p* < 0.05.

We conducted a similar analysis for individual harm considered as a motive for withdrawal, while controlling for societal harm as the motive of withdrawal. All assumptions were controlled and met, as previously described. To some extent, the results of this analysis mirrored the previous results. We found that being female (*B* = 0.07), having a partner (*B* = 0.05), a higher education status (*B* = 0.07), being a blood donor (*B* = 0.05), not being an organ donor (*B* = −0.08), a lower SVO score (*B* = −0.04) and a higher level of social trust (*B* = 0.07) were significant predictors of individual harm motives for withdrawal. There were no associations with the degree to which perceptions of individual or societal benefits motivated respondents’ participation in the biobank. The total explained variance was 17%.

### Willingness of respondents to allow their data to be linked

We attempted to predict participants’ intentions of sharing diverse personal data through linkages to medical research records according to participants’ demographic variables, motives for participation and withdrawal and prosocial psychological characteristics. All assumptions for this linear multiple regression analysis were met as well: the normality probability plot and the scatterplot of standardised residuals showed that the data met the assumptions of normality, linearity and homoscedasticity; there were no indications of multicollinearity (VIF scores <10.0; tolerance >0.1). Several significant predictors of the participants’ willingness to link their data to large-scale datasets for use in medical research were identified. These predictors were low levels of concern regarding potential harm relating to participation (*B* (Societal) = –0.08, *B* (Individual) = –0.10), a strong motivation to realise benefits via participation (*B* (Societal) = 0.20, *B* (Individual) = 0.05), a lower age (*B* = −0.13), being male (*B* = −0.07), a higher educational level (*B* = 0.09), being an organ donor (*B* = 0.07), a high level of trust in society (*B* = 0.09), trust in the government’s DM&H (*B* = 0.08), trust in commercial enterprises’ DM&H (*B* = 0.14) and trust in commercial researchers’ DM&H (*B* = 0.06). The total explained variance was 21%.

## Discussion

We investigated respondents’ motives for participating and potentially withdrawing their participation in a large-scale, centralised data repository for supporting scientific research in the Netherlands. In addition, we investigated associations between their motives for participation and (potential) withdrawal, respectively, and their demographic and psychological characteristics as well as their willingness to permit their personal data to be linked with a population-based biobank. Our results indicate that participants’ motives for both participation and potential withdrawal were clearly premised on societal and individual consequences. Whereas motives for participation were related to individual and societal benefits, motives for withdrawal were based on perceptions of potential societal or individual harm associated with participation in the biobank.

In line with previous studies, we found that both participation and (potential) withdrawal were primarily driven by societal motives [19, 24]. However, our findings about participation and withdrawal, which were based on assumptions of individual benefit and harm, respectively, suggest that individual motives for participation, especially those relating to potential harm, cannot be neglected. For example, highly educated participants were more likely to consider withdrawal because of potential individual harm than those who were less well-educated. Conversely, less well-educated participants were more likely to consider individual benefits when deciding to participate than highly educated participants. These findings confirm the results of a review study about motives to enrol in population-based biobank studies, which indicated the importance of consequences on an individual level [14]. Recent studies also found that predictors of individuals’ willingness to contribute genetic data related to their positive evaluations of genetic data for the benefit of society and themselves [37, 48].

In our model, trust was evidently a relevant predictor of motives for withdrawal. While these findings confirm those of previous studies regarding the importance of trust in biobank participation decisions [6, 21, 24], they also highlight the complexity of trust, especially in relation to withdrawal decisions. Anticipated societal harm as a motive for withdrawal associated with a lack of trust in society and in commercial enterprises and researchers, whereas anticipated harm to individuals motivating withdrawal decisions associated with a high level of trust in society. We might explain this counterintuitive finding as an indication of trust reducing perceived risks or anticipated harm for society when this trust is directed towards society including commercial enterprises. Individual harm motives, such as having a negative experience or cost of time, became more important when societal trust was high. In contrast, motives of anticipated societal harm were higher when trust is merely directed to government’ data management and handling.

Although there is strong evidence that trust is positively associated with stronger intentions to participate [6, 20], our findings support a view of trust as a conditional and fluid concept [6]. This is in line with recent results of an Anglo-Saxon study that showed a robust variability among trust categories in society, in particular the health care context [21]. Our results fit a definition of trust in society as a resource to cope with uncertainties [49]. Involvement of commercial enterprises in research, for example, is previously found to be a robust trigger for concerns of potential commodification or privacy violation which in combination with lack of trust led to withdrawal. These concerns touch upon current developments in biobanking [18]. Broad societal trust, including trust in commercial enterprises or researchers, might be a resource to cope with uncertainties in participation. Narrow societal trust—only directed on government behaviour towards data—is a limited resource. A limited resource is likely to draw attention away from pragmatic concerns on an individual level. Accordingly, higher levels of trust do not necessarily hinder decisions to withdraw from participation.

Furthermore, we found that participants’ motives were associated with several other psychological characteristics. Motives relating to societal benefits or societal harm were associated with stronger prosocial indicators, such as donor status, healthspheric or biospheric values and SVO, confirming the findings of previous studies that highlighted the importance of general attitudes regarding perceptions of the risks and especially the benefits of participation in a large-scale, centralised data repository for medical research [14, 33]. On top of that, several demographic factors, such as lower educational levels, being male or not being an organ donor, were predictors of stronger motives to pursue individual benefits via participation. By contrast, both individual and societal harm were important motives for women in potential decisions to withdraw participation. The same we found for blood donors in relation to individual harm, and in relation to societal harm motives for older participants and participants with a higher education status.

These findings suggest that gender, donor status, age and education play different roles in participation and withdrawal in a biobanking context. Being female, blood donor, older or well-educated may increase the likelihood of an individual’s withdrawal during changes, but these characteristics may also be positively linked to stronger perceptions of the benefits of medical research, greater comfort with giving blood and trust in the biobank [24]. Our results partly support studies showing that females are stronger oriented towards relational or altruistic goals, while men are oriented on their own or own group goals in participation decisions [23, 50]. Yet they emphasise results of a recent study about gender, age and educational level differences, which showed that these differences associated inconsistently with volunteering when taking into account context or other demographic and prosocial intrapersonal characteristics, such as age, marital status, values and societal trust [51]. An Australian study about biobanking confirmed these complexities in biobanking, since individuals with low educational levels are reportedly more willing to participate in biobanks that share resources or collaborate with commercial researchers than those who do not. Men were found to have more trust in biobanks collaborating with international researchers, while women had more trust in biobanks collaborating with national researchers [30]. Experimental designs in future studies seem to be worthwhile in light of these findings, since these designs, such as randomised controlled studies, are better able to distinguish robust associations, and conditional associations between participation/non-participation and characteristics. For example, these designs could unravel the unique effects of blood and organ donor status.

Our study extends the focus of previous studies on key mechanisms of participation in biobanking through an investigation of the factors that influence the probability of individuals’ withdrawal and their willingness to accept innovations in data collection, for example, data linking. The drivers of participants’ willingness to accept data linkages for medical research include a strong motivation to realise individual and especially societal benefits via participation, weak concerns about possible individual or societal harm and high levels of societal trust and trust in the whole context of research. This finding supports those of previous studies indicating the importance of perceived benefits, risks and trust [21, 33]. In addition, we found greater willingness to accept data linkages among young, male and well-educated participants. Some studies have found as well that men, younger people and higher educated people are more confident about participation than women, older people and lower educated people, for example, by perceiving fewer risks relating to genetic technologies [20, 33], perceiving more genetic exceptionalism [20] or having a broad societal trust [21].

Using data linkage for enriching data repositories could provide new opportunities for successfully obtaining more data of young, male and well-educated participants in biobanks. Furthermore, data repositories might recruit more young people and men when offering data linkages possibilities as these groups are otherwise less likely than others to participate in research. The prospect of data linkage might be more attractive for them, because of the lower effort participation brings. Another reason could be that the potential to find interesting new information pertains to these individuals. However, our results also showed that women, older people, and those with a lower education status are less inclined to contribute their personal information via data linkages, thus indicating and confirming the need for tailored recruitment or retainment strategies based on these characteristics [36]. Our findings may indicate that new information technology solutions can only partly be a solution for recruitment and retainment of participants, as more data isn’t synonymous with better research data. Further research could focus on investigating the factors that motivate these groups to be less or more inclined towards data-linking practices, and to unravel the variety of conditions for acceptance of data-linking practices.

More generally, whereas some groups within the population may be easier to recruit, other groups may be easier to retain when contextual changes in biobanking occur. These findings raise new questions about the current strict ideals on research ethics. For example, the ideal not to coerce individuals into participation in research with potential individual benefits, or to traverse the line of ‘safe’ contributions towards build-up of knowledge as a common good. As certain characteristics associate differently with motives and levels of trust depending on the context, a conservative approach on research ethics might have negative consequences for equal chances to participate in data repositories. Simultaneously, participation in data repositories might have more implications in the prospect of learning health systems [52], though the challenges of data security remain or are likely to increase [1].

A more progressive approach on reciprocity in biobank participation could accept and take into account populations’ diversity in trust and values about benefits of research, especially for groups based on gender or age. By focusing on how to proactively deal with these differences in recruiting and retention of participation, it may be possible to prevent collisions of principles of research ethics in a fundamental way [18]. For example, prevention of potential discrimination of certain groups of individuals in policies seems to deny these individuals being appropriately represented in research. Concepts of equity or justice will become more salient in innovative data repository and information technologies that aim to optimise participation and data density rates [3]. As these are being built, it is primarily a societal question how to adapt to their uncertainties and effects [1]. That is why adaptive and sustainable systems for safeguarding research participants and non-participants from harm should concurrently be discussed and developed, for example, with harm mitigation bodies [53]. These systems require to take the national and cultural context into account, such as levels of trust among a particular society or important values for populations in society [54]. By doing so, trust in these systems might be retained during uncertain contexts characterised by change, for example, increasing concerns about potential commodification, privacy violations and data security. The importance of the national and cultural context robustly explained differences in decision making about (non)participation both global [55] and within Europe [48]. Further research should aim to provide more evidence on the factors that increase participants’ concerns and potential withdrawal in relation to their perceptions of impact of (non)participation on future policies or treatment based on research. Especially, interaction between different characteristics and motives merit further investigation.

Although our study yielded insights into the motives behind participation and withdrawal of participation in biobanks, it had some limitations. All of the respondents were participants in a particular Dutch population-based biobank. Therefore, our findings may to some extent be specific to the Dutch societal context, population-based biobanking and prospective cohort studies. For example, distinctions between the public and private sectors may vary across countries as well as engagement in biobanking or concerns about privacy [6, 48]. In addition to that, our participants group could differ substantially in demographic and prosocial characteristics from those who have not or not yet participated [25]. These differences may apply as well to samples of different types of biobanks and cohort studies. Furthermore, causal effects are difficult to capture through exploratory cross-sectional online surveys. This issue could be resolved by using more experimental designs in future studies and qualitative investigations of these processes. Although our findings were statistically reliable, the models’ performances were relatively weak in explaining variance. Our instruments are tailored to our research question using partly validated measures and subscales, which might limit the generalisation and validity of our results. Thus, while our findings are illuminating to some extent, the mechanisms underlying participation and withdrawal require further elucidation. Nevertheless, our findings may provide valuable insights for developing effective recruitment strategies, data collection methods or data repositories.

## Conclusion

We have shown that motives for participation in and potential withdrawal of participation from a biobank can be differentiated into those relating to societal benefits and harm on the one hand and those relating to individual benefits and harm on the other hand. In addition, these motives are differently associated with participants’ demographic and intrapersonal characteristics. Our data suggest that the tendency to withdraw from participation could be countered by inducing perceptions of more individual benefits in case of perceptions of limited societal benefits and low narrow levels of trust in society. Yet the results emphasise the complexity and potential trade-offs in perceived harms for others. Our findings may contribute to improving recruitment and retainment strategies for large-scale medical data collection and eliciting participants’ agreement with data linkage by incorporating relevant values and highlighting the important benefits of the research for individuals.

## Supplementary information

Video summary

## Data Availability

The data that support the findings of this study are available from Lifelines biobank but restrictions apply to the availability of these data, which were used under license for the current study, and so are not publicly available. Data are however available from the authors upon reasonable request and with permission of Lifelines.
